# Rule Discovery in Milk Content towards Mastitis Diagnosis: Dealing with Farm Heterogeneity over Multiple Years through Classification Based on Associations

**DOI:** 10.3390/ani11061638

**Published:** 2021-06-01

**Authors:** Esmaeil Ebrahimie, Manijeh Mohammadi-Dehcheshmeh, Richard Laven, Kiro Risto Petrovski

**Affiliations:** 1 Genomics Research Platform, School of Life Sciences, College of Science, Health and Engineering, La Trobe University, Melbourne, VIC 3086, Australia; 2 Australian Centre for Antimicrobial Resistance Ecology, School of Animal and Veterinary Sciences, The University of Adelaide, Adelaide, SA 5371, Australia; manijeh.mohammadidehcheshmeh@adelaide.edu.au; 3 School of BioSciences, The University of Melbourne, Melbourne, VIC 3010, Australia; 4 School of Veterinary Science, Massey University, Auckland 0745, New Zealand; R.Laven@massey.ac.nz; 5 Davies Research Centre, School of Animal and Veterinary Sciences, The University of Adelaide, Adelaide, SA 5371, Australia

**Keywords:** farm heterogeneity, farm management, invisible mastitis, machine learning, meta-analysis, milking parameters, subclinical mastitis

## Abstract

**Simple Summary:**

Invisible (subclinical) mastitis decreases milk quality and production. Invisible mastitis is linked to an increased use of antimicrobials. The risk of the emergence of antimicrobial-resistant bacteria is a major public health concern worldwide. Therefore, early detection of infected cows is of great importance. Machine learning has opened a new avenue for early mastitis prediction based on simple and accessible milking parameters, such as milk volume, fat, protein, lactose, electrical conductivity (EC), milking time, and milking peak flow. However, farm heterogeneity is a major challenge where multiple patterns can predict mastitis. Here, we employed a classification based on associations and scaling approach for multiple pattern discovery over multiple years. The approach we have developed helps to address farm heterogeneity and generalise machine learning-based diagnosis of mastitis worldwide.

**Abstract:**

Subclinical mastitis, an economically challenging disease of dairy cattle, is associated with an increased use of antimicrobials which reduces milk quantity and quality. It is more common than clinical mastitis and far more difficult to detect. Recently, much attention has been paid to the development of machine-learning expert systems for early detection of subclinical mastitis from milking features. However, differences between animals within a farm as well as between farms, particularly across multiple years, are major obstacles to the generalisation of machine learning models. Here, for the first time, we integrated scaling by quartiling with classification based on associations in a multi-year study to deal with farm heterogeneity by discovery of multiple patterns towards mastitis. The data were obtained from one farm comprising Holstein Friesian cows in Ongaonga, New Zealand, using an electronic automated monitoring system. The data collection was repeated annually over 3 consecutive years. Some discovered rules, such as when the milking peak flow is low, electrical conductivity (EC) of milk is low, milk lactose is low, milk fat is high, and milk volume is low, the cow has subclinical mastitis, reached high confidence (>70%) in multiple years. On averages, over 3 years, low level of milk lactose and high value of milk EC were part of 93% and 83.8% of all subclinical mastitis detecting rules, offering a reproducible pattern of subclinical mastitis detection. The scaled year-independent combinational rules provide an easy-to-apply and cost-effective machine-learning expert system for early detection of hidden mastitis using milking parameters.

## 1. Introduction

Inflammation of the bovine udder, known as mastitis, is the most significant disease of dairy cattle worldwide, as well as a major public health concern. It is usually caused by bacterial infections [1]. Worldwide, the economic losses due to mastitis are estimated to be approximately EUR 100 per cow for each farm per year, as mastitis reduces both milk quality and quantity [1,2]. Furthermore, treatment of mastitis is the main reason for the use of antimicrobials on many dairy farms in the US [3]. Thus, higher mastitis prevalence increases antimicrobial use and, consequently, the risk of developing antimicrobial resistance.

Mastitis can be divided into clinical (visible signs of inflammation of the udder or milk) and subclinical (inflammation without visible signs). Subclinical mastitis is much more common than clinical mastitis [4,5]. Detection of subclinical mastitis at an early stage is important in preventing spreading of infection to other cows, maintaining high milk quality, and enabling early and effective treatment of affected cows. Early detection of subclinical mastitis needs effective and accurate active surveillance strategies. Daily measurement of milk somatic cell count, called test-day somatic cell count, is the most used method for detecting subclinical mastitis at the individual cow level. However, somatic cell count widely fluctuates between test days. Furthermore, test-day somatic cell count is a late indicator, where the inflammation is already evident. However, there is an increasing availability of precision technologies on farms, such as in-line detectors, which record milk quality parameters, milking characteristics, as well as somatic cell counts, providing a new source of big data sets for subclinical mastitis diagnosis.

The use of somatic cell count-independent predictors, such as milk quality parameters, especially if combined with longitudinal monitoring of somatic cell count could increase the robustness and early predictive power of subclinical mastitis diagnosis. One potential means of further enhancing such testing could be the use of machine-learning expert systems to develop mathematical models of subclinical mastitis occurrence using milking variables. Measurement of simple and accessible milking parameters, such as milk volume, fat, protein, lactose, electrical conductivity (EC), milking time, and milking peak flow, all of which are, or can be, measured on dairy farms equipped with precision technologies, is an ideal source of data input for machine learning systems designed for early detection of subclinical mastitis. A variety of machine learning models have been employed for mining of milking parameters for prediction of subclinical mastitis. Random Forest, Deep Learning, and Gradient-Boosted Trees models showed high performance in pattern recognition in milking parameters towards subclinical mastitis [4,6].

The next step is expansion of models from a single/limited number of farms to many farms and over multiple years, called generalisation of machine-learning based expert system. Animal and farm level variability, as well as variability over time, are all sources of heterogeneity which need to be considered as part of the process of globalization of machine learning models. Farm heterogeneity is a major challenge where predictive parameters of subclinical mastitis diagnosis can vary from one animal to another. Furthermore, the cut-off of milking predictors can change on a year-to-year or farm-to-farm basis, due to change in a variety of factors such as weather, farm husbandry and management, and diet regime.

Reducing heterogeneity across samples (batch effects) using Z-standardisation or scaling is an essential step in the generalisation of results and meta-analyses [7,8,9,10]. Quartiling, which categorises values of each feature into four bins (Q1, Q2, Q3, and Q4) outperforms the other scaling and standardization methods [8] and reduces batch effects where ranges (cut-offs) of a quartile are different across years and farms. Heterogeneity-reduction using quartiling has been employed extensively for multiple pattern discovery in large-scale datasets, particularly in combination with the classification based on associations (CBA) model [8,11,12,13]. CBA provides the chance to mine completely scaled data as categorical/polynomial variables. It is an integrated classification and association rule mining algorithm that utilises the qualities of both classification and association rules and can be applied to analyse complex data [11,14]. The CBA algorithm can reveal valuable rules that existing classification systems fail to discover [11,15,16]. CBA does not build a single model. In contrast, CBA generates numbers of rules (similar to sub-models) to characterise subgroups in big and complex data sets. CBA is a combined algorithm with the strengths of both classification and association rule mining through class association rules (CARs) [16]. Further improvements have elaborated the rule discovery efficiency of the model, particularly in analysis of unbalanced data, like mastitis data [17,18,19]. Classification based on associations is an accurate classifier for prediction. Furthermore, the model can mine association rules. While there are obvious benefits in exploring such a combinational data mining approach, to date no study has applied CBA in the field of subclinical mastitis.

Combining scaling-based meta-analysis and CBA could provide a means of removing farm heterogeneity when predicting subclinical mastitis. The aim of this study was to develop an integrated pipeline of data quartiling and CBA for multiple patterns discovery over multiple years.

## 2. Materials and Methods

**Figure 1:**
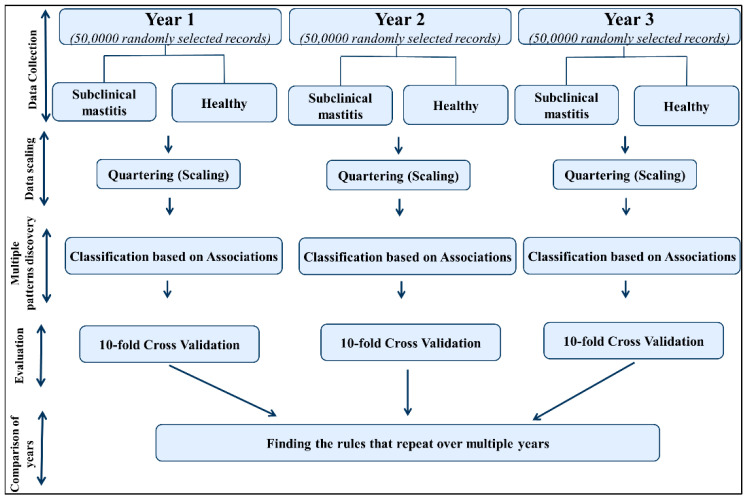
Schematic representation of the rule discovery pipeline for subclinical mastitis prediction from milking features over multiple years by combination of data scaling (quartiling) and classification based on associations model.

Figure 1 represents the schematic workflow of the developed rule discovery pipeline.

### 2.1. Data Collection

Milking data were collected over 3 years (2011, 2012, and 2013) from a commercial dairy farm in Ongaonga, New Zealand. The herd was a block calving herd (July to October/March to May) of principally Holstein Friesian cows, milked twice daily through a rotary parlour. The average age for the herd was 6.7 years per cow. Data was collected at each milking for the following parameters: total volume (Vol), fat, protein (Prot), and lactose (Lact) concentration, electrical conductivity (EC), milking time, peak flow, and somatic cell count. Somatic cell count values ≥250,000 cells/mL of milk was used as the main indication of subclinical mastitis occurrence [20]. The unbalanced nature of data needs to be addressed in application of machine learning. The cows with clinical mastitis were milked through a separate milking parlour and were not included in the datasets.

### 2.2. Generation of Year-Based Quartiled/Polynomial Datasets

For each year, separately, the values of each measurement collected at milking (i.e., Vol, fat, protein, Lact, EC, milking time, and peak flow) were converted to nominal features by discretizing the data for each measurement into four bins of equal frequency: Q1, Q2, Q3, and Q4 (from lowest to highest). Quartiles to produce these four bins were automatically generated using a “Discretize by Frequency” operator of RapidMiner Studio software 9.8.001 (RapidMiner Inc., Boston, MA, USA). Consequently, a scaled/polynomial dataset of measurements was generated for each year. A binomial target variable (label) was added to the datasets, based on the above-mentioned somatic cell count cut-off (≥250,000 cells/mL = subclinical mastitis, <250,000 cells/mL = healthy). In addition to 250,000 cells/mL, the analysis was repeated with 3 different cut-offs of somatic cell count of 250,000 cells/mL, somatic cell count of 200,000 cells/mL, somatic cell count of 150,000 cells/mL, and somatic cell count of 100,000 cells/mL. In each year, 50,000 records were randomly selected for rule discovery using the classification based on associations model.

### 2.3. Rule Discovery in Scaled Data of Milking Features towards Subclinical Mastitis by Classification Based on Associations Model

The CBA model has 2 parts: (1) A rule generator for extracting association rules (CBA-RG algorithm), and (2) a classifier builder (CBA-CB), which builds a classifier using class-associated rules [11]. To produce the best classifier out of the entire set of rules, a minimum number of rule sets is selected to cover the training dataset and minimize the error rate [16]. Analysis was performed using CBA (v2.1), developed at the School of Computing, National University of Singapore [16]. The arulesCBA package is equivalent to a tool in R environment [21]. Performance criteria of accuracy, sensitivity, specificity, F_measure, precision, and AUC were calculated for CBA model, run on quartiled/scaled dataset in each year.

Modelling was performed for each year using all milking parameters (each of the 3 years analysed separately). The employed approach resulted in rule discovery in each year. Then, rules in different years were compared to uncover the most reproducible and robust predictive rules over multiple years. The analysis was repeated for each somatic cell count cut-offs 250,000 cells/mL, 200,000 cells/mL, 150,000 cells/mL, and 100,000 cells/mL.

### 2.4. Cross Validation to Estimate the Performance of the Developed Pipeline in Analysis of Future Unseen Data

Ten-fold cross validation was used to evaluate the statistical performance of the developed pipeline of the data scaling + CBA model in analysis of future data. The cross validation algorithm used had two parts: (1) a training subset for model development, and (2) testing subsets for measuring model performance. The process was repeated 10 times, called 10-fold cross validation, and the mean and standard deviation of accuracy on testing subsets in each year were calculated.

### 2.5. Statistical Analysis

To compare the prevalence of subclinical mastitis in each ranked bin for each measurement collected at milking, two-sample proportion tests, and Fisher Exact test were conducted. This analysis was performed using Minitab 19 Statistical Software (Minitab Inc., State College, PA, USA).

## 3. Results

### 3.1. Dealing with Farm Heterogeneity to Diagnose Subclinical Mastitis by Running Classification Based on Associations on the Ranked Datasets of Milking Features

Frequency of subclinical mastitis, based on somatic cell count values ≥250,000 cells/mL of milk, was 21% in year 1, 20% in year 2, and 24% in year 3. Table 1 represents statistics of data after removal of records (rows) with missing data.

**Figure 2:**
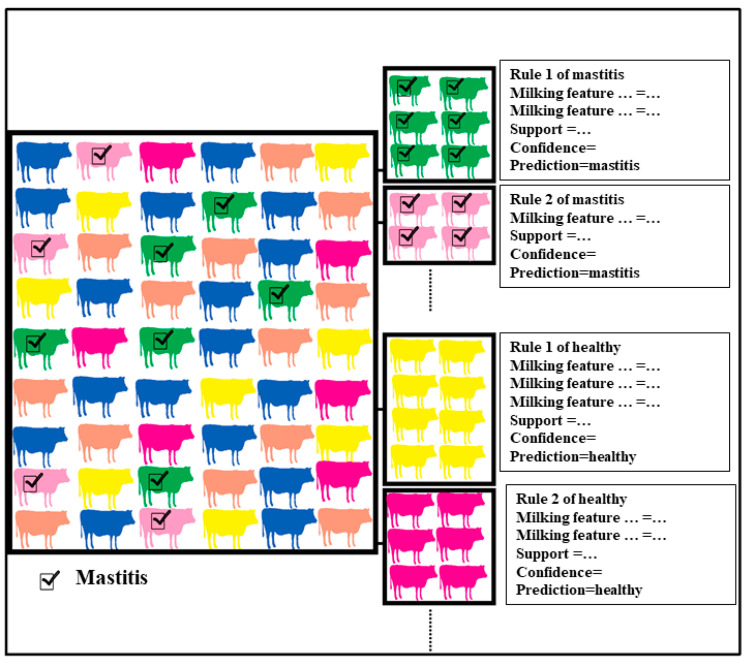
Classification based on associations is a high performing and robust classifier that integrates the classification algorithm with the association rule mining algorithm to deal with data heterogeneity. Classification based on associations finds homogenous groups within heterogenous data, based on minimum support. Then, classifier applies discriminative rules with high confidence in each homogenous group.

As presented in Figure 2, classification based on associations is a robust classifier that finds uniform subgroups within heterogenous data. Then, the model finds rules with high confidence for each uniform subgroup. An association rule has two parts, made up of “if” and “then”, to find a group of records within a heterogeneous dataset where a reliable rule can be applied with high confidence on those records.

The generated multiple predictive patterns provide the capability to analyse the mixed and heterogenous data. Classification based on associations can easily mine the polynomial/categorical data. This strength emphasises the suitability of this model in multiple years or multiple farms.

Association rule mining has two terms of support and confidence to represent the most important relationships (Figure 2). Support describes how frequently the items appear in the dataset, and confidence shows how the statement is likely to be true.

- Coverage (Cov) %: The percentage of records containing the conditions in data set.
- Cover Count: The number of records containing the conditions.
- Support (Sup) %: The percentage of records containing both conditions and class.
- Support Count (SupCount): The number of records containing both conditions and class.
- Confidence (Conf) %: The ratio of support to coverage. This shows the precision of the rule in the data set.

### 3.2. Discovered Rules of Subclinical Mastitis Prediction from the Quartiled Milking Features in the First Year

Supplementary S1 presents the discovered rules of subclinical mastitis/healthy prediction in year 1, based on somatic cell count cut-off of 250,000 cells/mL. The overall error of classification was 16.8 % (accuracy of 83.2%). The top five rules of subclinical mastitis prediction that reached to the high confidence of 72.4%, 69.7%, 69.4%, and 67.6%, respectively, are listed here:

- Rule 189: EC = Q4, Lact = Q1, Prot = Q4, Fat = Q4, Vol = Q1 -> class = Mastitis
  (Cover% = 0.24%, Conf% = 72.3%, CoverCount = 123, SupCount = 89, Sup% = 0.18%);
- Rule 190: Peak_Flow = Q1, EC = Q4, Lact = Q1, Prot = Q4, Fat = Q4 -> class = Mastitis
  (Cover% = 0.26%, Conf% = 69.8%, CoverCount = 129, SupCount = 90, Sup% = 0.18%);
- Rule 191: Peak_Flow = Q1, Milking_Time = Q1, EC = Q4, Lact = Q1, Fat= Q4, Vol = Q1 -> class = Mastitis
  (Cover% = 31%, Conf% = 69.4%, CoverCount = 157, SupCount = 109, Sup% = 0.22%);
- Rule 192: EC = Q4, Lact = Q1, Prot = Q4, Fat = Q4 -> class = Mastitis
  Cover% = 0.54%, Conf% = 67.66%, CoverCount = 269, SupCount = 182, Sup% = 0.36%);
- Rule 193: Peak_Flow = Q1, EC = Q4, Lact = Q1, Fat = Q4, Vol = Q1 -> class = Mastitis
  (Cover% = 0.64%, Conf% = 67.2%, CoverCount = 321, SupCount = 216, Sup% = 0.43%).

The highest confidence (72.3%) was obtained in Rule 189 with the top quartiles of EC, protein, and fat (Q4) combined with the lowest quartiles of lactose and milk volume (Q1). The coverage of this rule was 24%, applied to 123 records (cases) in year 1. In contrast, the highest coverage (0.64%) was obtained in Rule 193 with the lowest quartiles of Peak_Flow = Q1, lactose, and milk volume combined with the highest quartiles of milk EC and Fat (Q4).

### 3.3. Rules of Subclinical Mastitis Prediction from the Quartiled Milking Features in the Second Year

Rules of subclinical mastitis/healthy prediction at the second are presented at Supplementary S2. The overall accuracy of 83.8% (error of 16.2%) was obtained. The top rules of subclinical mastitis detection were:

- Rule 215: Peak_Flow = Q1, Milking_Time = Q1, EC = Q4, Lact = Q1, Prot = Q4, Vol = Q1 -> class = Mastitis
  (Cover% = 0.22%, Conf% = 77.3%, CoverCount = 110, SupCount = 85, Sup% = 0.17%);
- Rule 216: Peak_Flow = Q1, Milking_Time = Q3, EC = Q4, Lact = Q1, Fat= Q3, Vol = Q1 -> class = Mastitis
  (Cover% = 0.24%, Conf% = 73.96%, CoverCount = 119, SupCount = 88, Sup% = 0.18%);
- Rule 217: Peak_Flow = Q1, EC = Q4, Lact = Q1, Fat = Q4, Vol = Q1
  -> class = Mastitis
  (Cover% = 0.79%, Conf% = 72.5%, CoverCount = 397, SupCount = 288, Sup% = 0.58%);
- Rule 218: EC = Q4, Lact = Q1, Prot = Q4, Fat = Q4, Vol = Q1
  -> class = Mastitis
  (Cover% = 0.43%, Conf% = 69.3%, CoverCount = 215, SupCount = 149, Sup% = 0.30%);
- Rule 219: Peak_Flow = Q1, EC = Q4, Lact = Q1, Prot = Q4, Fat = Q4
  -> class = Mastitis
  (Cover% = 0.34%, Conf% = 69.2%, CoverCount = 169, SupCount = 117, Sup% = 0.23%).

The highest confidence (77.3%) in subclinical mastitis diagnosis was obtained in Rule 215 when Peak_Flow, Milking_Time, volume, and lactose content were at the lowest level (Q1) and EC and protein were at the highest level (Q4). In contrast, Rule 217 showed the highest coverage (397 records) where Peak_Flow = Q1, EC = Q4, lactose= Q1, fat = Q4 and volume = Q1.

### 3.4. Multiple Patterns Discovery towards Subclinical Mastitis Using the Quartiled Milking Features in the Third Year

Year 3 rules of subclinical mastitis/healthy prediction, derived from a combination of ranked (scaled) milking features, are presented in Supplementary S3 in year 3. The overall error of classification was 81.9%. The top five rules of subclinical mastitis occurrence were:

- Rule 207: Peak_Flow = Q1, Milking_Time = Q2, EC = Q4, Lact = Q1, Fat = Q4
  -> class = Mastitis
  (Cover% = 0.32%, Conf% = 79.6%, CoverCount = 162, SupCount = 129, Sup% = 0.26%);
- Rule 208: Peak_Flow = Q1, EC = Q4, Lact = Q1, Fat = Q4, Vol = Q1
  -> class = Mastitis
  (Cover% = 0.78%, Conf% = 79.5%, CoverCount = 392, SupCount = 312, Sup% = 0.624%);
- Rule 209: Peak_Flow = Q1, Milking_Time = Q1, Lact = Q1, Fat = Q4, Vol = Q1
  -> class = Mastitis
  (Cover% = 0.47%, Conf% = 77.3%, CoverCount = 238, SupCount = 184, Sup% = 0.36%);
- Rule 210: Peak_Flow = Q1, EC = Q4, Lact = Q1, Prot = Q3, Vol = Q1
  -> class = Mastitis
  (Cover% = 0.43%, Conf% = 76.5%, CoverCount = 217, SupCount = 166, Sup% = 0.33%);
- Rule 211: Peak_Flow = Q1, Milking_Time = Q1, EC = Q4, Lact = Q1, Prot = Q1, Vol = Q1
  -> class = Mastitis
  (Cover% = 0.38%, Conf% = 76.17%, CoverCount = 193, SupCount = 147, Sup% = 0.29%);
- Rule 212: Peak_Flow = Q1, EC = Q4, Prot = Q4, Vol = Q1
  -> class = Mastitis
  (Cover% = 0.49%, Conf% = 75.4%, CoverCount = 248, SupCount = 187, Sup% = 0.37%).

The lowest levels of Peak_Flow, Milking_Time, and lactose in combination with the highest levels of EC and fat = Q4 resulted in identification of subclinical mastitis with high confidence of 79.6% (Rule 207). High coverage of 392 records with notable confidence of 79.5% was achieved with the lowest levels of Peak_Flow, lactose, and volume ((Q1 quartile) combined with the highest values of EC and Fat (Q4).

### 3.5. Reproducible Rules of Subclinical Mastitis Diagnosis from Milking Features over Multiple Years

Subclinical mastitis identifying rule of Peak_Flow = Q1, EC = Q4, Lact = Q1, Fat = Q4, and Vol = Q1 was repeated over multiple years covering 321, 397, and 169 cases in year 1, year 2, and year 3, with high confidence of 67.2%, 72.5%, and 69.2%, respectively. Supplementary S4: Discovered rules of subclinical mastitis identification from scaled/quartiled datasets of milking parameters in multiple years. Year-based data scaling data quartiling provided the opportunity to compare the rules of multiple years.

### 3.6. Feature Selection Using Classification Based on Associations

Multiple appearances of a feature in many association rules, called feature overrepresentation/enrichment, is an index of feature importance [12,13]. Supplementary S4 provides an overview of quartiles of milking features that are important for identification of subclinical mastitis/healthy records. Table 2 summaries the key quartiles of milking features that repeat in rules of subclinical mastitis classification (over-represented quartiles) over multiple years.

First quartile (Q1) of milk lactose made up 85%, 100%, and 94.3% of subclinical mastitis detecting rules in Year 1, Year 2, and Year 3, respectively, with the average being 93.1% ± 7.5%. In contrast, top quartile (Q4) of EC was present in 100%, 100%, and 51.4% of subclinical mastitis detecting rules, with an average of 83.8% ± 28.1%. Q1 of lactose and Q4 of EC offer a reproducible pattern of subclinical mastitis detection over multiple years. Q1 of milk volume, Q1 of Peak_Flow, Q4 of protein, Q4 of fat, and Q1 of Milking_Time comprise the next level of predictors. Standard deviation provides a good index of repeatability of subclinical mastitis indicators over time. For example, standard deviation of Q4 level of fat content is 2.9%, while this value increases to 29.1% in protein content. This shows that Q4 of fat content is more stable and reproducible than Q4 of protein content.

### 3.7. Risk Quartiles of Subclinical Mastitis Diagnosis

**Figure 3:**
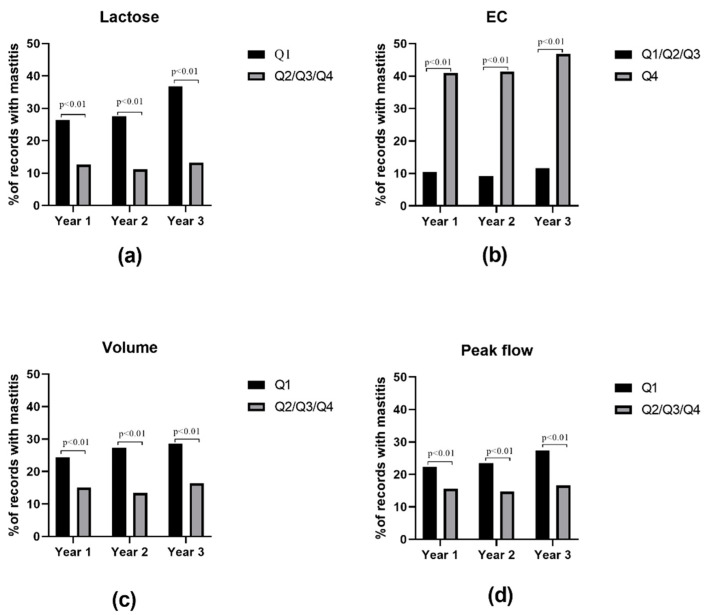
Quartile/scaled based risk factors of subclinical mastitis. (**a**) Lactose content of milk. (**b**) Electrical conductivity meter (EC) of milk. (**c**) Milk volume, and (**d**) milking peak flow. First quartiles of lactose, volume, and peak flow had significantly higher abundances of subclinical mastitis cases over multiple years. In contrast, in all 3 years, top quartile (Q4) of EC contained remarkedly higher abundances of subclinical mastitis cases. Proportion test based on Fisher Exact test was used for analysis. Risk quartiles were derived from classification based on associations analysis (please refer to Section 3.7.). Changing the somatic cell count cut-off of 100,000 cells/mL did not make a major difference in risk factors and predictive rules.

Figure 3 presents the results of proportion test on the risk quartiles of milking features, derived from Classification based on Associations rules. Changing the lactose content of milk from Q2/Q3/Q4 to Q1 significantly (*p* < 0.01) increases the subclinical mastitis risk from 12.5% to 26.4% (2.1 fold increase) in year 1, from 11.2% to 27.5% (2.4 fold increase) in year 2, and from 13.2% to 36.9% (2.8 fold increase) in year 3.

Compared to Q1/Q2/Q3, EC in Q4 level significantly (*p* < 0.01) increases the risk of subclinical mastitis, from 10.4% to 41% increase (3.9 fold increase) in year 1, from 9.2% to 41.3% increase (4.5 fold increase) in year 2, and from 11.6% to 46.8% increase (4.1 fold increase) in year 3.

Decline in milk volume or peak flow to Q1 resulted in an increased chance of subclinical mastitis between 1.4–2 times in different years.

### 3.8. Ten-Fold cross Validation Analysis Showed Stablility of Classification Based on Associations-Derived Rules

According to 10-fold cross validation, the pipeline reached an overall accuracy of 83.2% in year 1, 83.8% in year 2, and 81.85% in year 3 (Table 3). The variation between folds of cross-validation was low (low standard deviation). Low standard deviations between folds of cross validation demonstrate the robustness of the model in dealing with new data.

## 4. Discussion

Management of dairy farms through machine learning-based analysis of milking features has received increased attention in recent years [4,6,8,22,23,24,25,26,27,28]. The next step is globalisation of a machine-learning based expert system from milking parameters. However, animal differences within a farm as well as between the farms, particularly in multiple years, need to be considered first. In practice, to generalise the findings, it is crucial that the recommended models address the following concerns: (1) Within farm variability, (2) year-to-year variability, and (3) farm-to-farm variability. In this study, we developed a pipeline for reducing data heterogeneity (batch effects) by a combination of data quartiling and classification based on associations model. Integration of scaling with classification based on associations removed the year (batch) effect and found the best combination of milking features in quartile terms for predicting subclinical mastitis.

Quartiling (scaling) helps generalisation of models where cut-off/range of the same quartile can change between years/farms [7,8,9,10]. Suppose that the overall values of milk volume, lactose, protein, and fat are up to 3-fold different between two farms or on one farm across different years, due to differences in feeding regime, breed, farm management, etc. It is not possible to apply a numerical cut-off from the first farm to the second farm for mastitis prediction from milking features. However, in farm-based scaling, the value for each farm converts to quartile values of Q1, Q2, Q3, and Q4 first, providing the opportunity to apply the numerical-independent cut-offs from the first farm to the second one. Scaled-based correlation, called rank of correlation coefficient, has shown higher performance in meta-analysis of expression data and integration of different experiments [29]. The scaled year-independent combinational rules provide an easy-to-apply and cost-effective machine-learning expert system for early detection of hidden mastitis using milking parameters. In this study, instead of numerical cut-off, we introduced quartile-based risk factors, including Q1 of lactose content, milk volume, and peak-flow combined with Q4 of EC. In our previous studies, we found that milk lactose and EC are potential predictors for subclinical mastitis [30,31] and has been confirmed in subsequent studies on Austrian Fleckvieh cows [31], Italian water buffaloes [32], Fleckvieh cattle [33], and goats [34]. EC level goes up during mastitis due to an increase in Na + and Cl- and a decrease in other mineral substances [20].

Multiple rule discovery using the classification based on associations approach provides the opportunity of prediction at the animal level through multiple patterns. In other words, different predictive rules can be assigned to two animals in a similar farm. Classification based on Associations and subgroup discovery have been employed for multiple rule discovery in heterogeneous large-scale data such as signature of increased host range and outbreak of influenza [11,12,13] or student course performance [10,35]. Some discovered rules, such as when the Peak_Flow = Q1, EC of milk = Q4, lactose = Q1, fat = Q4, and volume = Q1 the cow has subclinical mastitis in the current study and reached a high confidence (>70%) in multiple years.

Choice of somatic cell count cut-off for subclinical mastitis is important in predictive model development. Different cut-offs for subclinical mastitis are selected in previous studies, such as 250,000 cells/mL, 200,000 cells/mL, 150,000 cells/mL, and 100,000 cells/mL [20,36,37]. In this study, cows with clinical mastitis were milked through a separate milking parlour and were not included in the data. In our previous work [20], we compared the three cut-offs 150,000 cells/mL, 200,000 cells/mL, and 250,000 cells/mL for subclinical mastitis. The milking risk factors were similar for all three cut-offs [20]. In this study, changing the cut-off did not change the risk factors and model performance that demonstrates the robustness of the discovered rules in this study.

Classification based on associations model is an ensemble learning method for classification and rule discovery with the ability to analyse categorical/polynomial features such as quartiles. Low variation between folds of 10-fold cross-validation was noticeable. Cross-validation has a nested structure and can estimate how accurately a model functions in practice. Low variation has been observed in the other ensemble learning models such as random forests, where decision is based on voting of multiple models rather than a single model. In the random forest approach, a multitude of decision trees (commonly in a range of 10–100) are constructed. In the classification based on associations approach, multiple rules predict the target variable (label). Ensemble learning models, such as classification based on associations, are especially beneficial when the data is heterogeneous, which is inevitable when we are using data from multiple different farms or multiple time-points of data collection. One of the main challenges in analysis of unbalance data such as mastitis is the unbalanced nature of data, where there are many more healthy cases than diseased cases in a population. Due to higher number of healthy cases for model training, the model receives better training for healthy cases than diseased ones. Consequently, sensitivity for mastitis detection drops [6]. Balancing data has been used in this case to train the model for healthy and mastitis cases with similar precision [4,20]. However, in practice, a machine learning expert system should be faced with a highly unbalanced data. Classification based on Associations can deal with this obstacle by finding uniform subgroups within data and applying rules with high precision and confidence in each subgroup. In other words, while the overall sensitivity of dataset is low, classification based on associations gives rules with high sensitivity, covered a proportion of dataset.

Prediction of subclinical mastitis is an important management tool for dairy farms. The developed rule discovery pipeline in this study is easily applicable in farm management systems and requires the following tools: RapidMiner Studio software, and either CBA (v2.1) software or arulesCBA R package. A limitation of this study was that the lactation number was not recorded at the time of data collection. Incorporating of lactation parameter in future studies/applications can improve the prediction power of the developed pipeline. If mastitis can be accurately predicted management changes can be implemented (e.g., increase in teat disinfection concentration, diet manipulations) and mastitis episodes prevented. Currently, machine learning and methodologies described in the current study can be applied only to farms with appropriate regular monitoring of milking parameters. As dairy farms become globally more and more sophisticated, this limitation of the current study will become obsolete.

## 5. Conclusions

In this study, for the first time, integration of CBA and data scaling resulted in the discovery of multiple reproducible rules of subclinical mastitis occurrence measured over multiple years using various milking parameters. The scaled year-independent combinational rules provide an easy-to-apply and cost-effective machine-learning expert system for early detection of hidden mastitis using milking parameters. Applying models such as classification based on associations that account for the mixed and heterogenous nature of farm data elaborated by employing the ranked/scaled features provides the opportunity to find accurate predictive indicators of farm management, independent from year or farm. Classification based on associations works on polynomial data, and it is possible to add/include the additional categorical variables such as farm-type, diet feeding regime, or breed. The mentioned strengths emphasise the suitability of Classification based on Associations in farm management. The developed approach helps to address farm heterogeneity and generalise machine learning based diagnosis of subclinical mastitis.

## Figures and Tables

**Table 1:** Number of healthy and subclinical mastitis records, collected over 3 years during the study.

Year	Healthy Records	Subclinical Mastitis Records (Somatic Cell Count Cut-Off of ≥250,000 cells/mL)
Year 1	57,180	12,034
Year 2	118,126	24,218
Year 3	68,789	16,657

**Table 2:** Quartiles of milking features that repeat in rules of subclinical mastitis classification (over-represented quartiles) over multiple years.

Year	Mastitis Rules no.	Number and Percentage of Subclinical Mastitis-Detecting Rules across Years 1, 2, and 3.						
		Lactose_Q1 ^1^	EC_Q4	Volume_Q1	Peak_Flow_Q1	Protein_Q4	Fat_Q4	Milking_Time_Q1
Year 1	20	17 (85%)	20 (100%)	9 (45%)	11 (55%)	7 (35%)	9 (45%)	5 (25%)
Year 2	10	10 (100%)	10 (100%)	6 (60%)	5 (50%)	8 (80%)	4(40%)	2 (20%)
Year 3	35	33 (94.3%)	18 (51.4%)	24 (68.6%)	15 (42.8%)	9 (25.4%)	14 (40%)	10 (28.6%)
Ave. % ± SD		93.1% ± 7.5%	83.8% ± 28.1%	57.9% ± 11.9%	49.27% ± 6.1%	46.8% ± 29.1%	41.7% ± 2.9%	24.5% ± 4.3%

^1^ Q stands for quartile. Q1 contained 25th percentile of milking feature with the lowest values. Q4 covered 25th percentile of milking feature with the highest values.

**Table 3:** Overall error of classification based on associations in subclinical mastitis prediction, based on 10-foldcross-validation analysis.

Year 1	Year 2	Year 3
17.9	16.6	19.7
17.1	17.1	19.8
17.8	17.0	19.5
17.9	17.4	19.9
17.1	16.7	19.1
17.7	17.1	18.9
17.1	17.2	19.0
17.8	16.5	19.6
17.5	17.4	18.5
16.7	16.5	20.6
Avg 17.4 ± 0.4	Avg 17.4 ± 0.4	Avg 19.4 ± 0.6

## Data Availability

The data is publicly available at CloudStor, aarnet, Australia, at the following link: https://cloudstor.aarnet.edu.au/plus/s/FkvQHff5HjQFtbA.

## Supplementary Materials

The following are available online at https://www.mdpi.com/article/10.3390/ani11061638/s1, Supplementary S1: Discovered rules of subclinical mastitis prediction in year 1, Supplementary S2: Discovered rules of subclinical mastitis prediction in year 2. Supplementary S3: Discovered rules of subclinical mastitis prediction in year 1. Supplementary S4: Discovered rules of subclinical mastitis identification from scaled/quartiled datasets of milking parameters in multiple years.
